# Multiple Confabulations Found in Bioinformatics Tasks Carried Out by Several Free Large Language Models

**DOI:** 10.2174/0113892029403403260112075225

**Published:** 2026-03-19

**Authors:** Diego A. Forero

**Affiliations:** 1 School of Health and Sport Sciences, Fundación Universitaria del Área Andina, Bogotá, Colombia

**Keywords:** Bioinformatics, computational genomics, large language models, generative artificial intelligence

## Abstract

Bioinformatics is one of the main areas in the health sciences with great potential for the application of Large Language Models (LLMs), as it mainly involves computational analysis of results derived from experimental and high-throughput analysis of human, animal, and cellular models. Testing available general-purpose LLMs for the presence of inaccurate answers, such as confabulations, is of great interest in the fields of bioinformatics and computational genomics. In the current study, we carried out an analysis of the performance of six freely available LLMs (Gemini, ChatGPT, Grok, Claude, Llama, and DeepSeek) in a number of tasks commonly used in bioinformatics and computational genomics, with varying levels of difficulty. The selected tasks were: converting different identifiers (for two organisms), simulating a bisulfite conversion of DNA sequences, identifying the effects on amino acids of DNA polymorphisms, retrieving orthologues in mouse, identifying GO ontologies and KEGG pathways for lists of genes, interpreting a Volcano plot for gene expression, and automatically generating R code to visualize data. In general, our analysis identified a multiplicity of confabulations for different types of results generated by the tested LLMs. Our results highlighted a high number of errors in the output of the LLMs and identified automatic generation of code as a promising area. Future studies will be needed for a better understanding of the causes of confabulations of LLMs in research-related tasks.

## 1 INTRODUCTION

Generative Artificial Intelligence (genAI), particularly Large Language Models (LLMs) [1], has been proposed very recently as a potential major breakthrough in multiple areas [2]. In this context, in the fields of health sciences and biomedical research, there is a need for detailed comparison and validation, from an independent and neutral perspective, of the results generated by the available LLMs [1-6].

Bioinformatics is one of the main areas in the health sciences [7-10] with a great potential for the application of LLMs [11-17], as it mainly involves computational analysis of results derived from experimental and high-throughput analyses carried out in humans and in animal and cellular models [18-21]. As in other fields of research related to human health, it is of paramount importance that scientists use validated tools to generate high-quality data (with a very low rate of inaccurate information) [22]. In this context, testing available general-purpose LLMs for the presence of inaccurate answers, such as confabulations, is of great interest in the fields of bioinformatics and computational genomics [23, 24].

Bioinformatics involves multiple tasks, such as conversion of IDs, manipulation of DNA sequences, functional enrichment analyses, and handling of large datasets, among others [18, 20]. Of particular relevance for this work, bioinformatics has an important tradition in terms of the use of free tools for its analyses of high-throughput data [18].

In the current study, an analysis of the performance of six freely available LLMs on a number of tasks commonly used in bioinformatics and computational genomics, with varying levels of difficulty, was carried out, and multiple types of frequent confabulations in those results were identified.

## 2 METHODS

Freely available, multi-purpose LLMs were identified in review articles and preprints about recent advances in genAI [1, 25, 26]. Free versions of Gemini [27], ChatGPT [28], Grok [29], Claude [26], Llama [30], and DeepSeek [31] were included in this study. An overview of the six freely available LLMs tested in this work is shown in Table **1**. For some LLMs, at the time of carrying out this study (January 2025), it was not possible to include figures as input (some of them only allow for text as input). As the focus was on LLMs that are very user-friendly (those with an easy-to-use web interface), recent LLMs designed for bioinformatics, such as AutoBA [32], BioMANIA [33], or BioMaster [34], were not tested, as they need to be downloaded from GitHub and require complex installation and deployment procedures.

A number of tasks commonly used in bioinformatics and computational genomics, with varying levels of difficulty, were selected: conversion of different IDs (for two organisms), simulation of a bisulfite conversion of DNA sequences, identification of the effects of DNA polymorphisms on amino acids, retrieval of orthologues in mouse, identification of GO ontologies and KEGG pathways for lists of genes, interpretation of a Volcano plot for gene expression, and automatic generation of R code to visualize data. In general, as a standard for comparison, we contrasted the outputs of the LLMs with those of state-of-the-art computational genomics tools [35-38], which have been designed, validated, and widely used for the respective tasks [18].

Randomly selected lists of genes were selected as inputs for the conversion of IDs: human gene symbols to Ensembl gene IDs, human Affymetrix probe IDs to gene symbols, and mouse gene symbols to Ensembl gene IDs. The DAVID [35], g: Profiler [36], and Ensembl BioMart [38] online tools were used to retrieve the correct lists of ID conversions, and a consensus was manually verified by a human expert.

For the study of manipulations and interpretation of changes of short genetic sequences, random DNA sequences were used. The six LLMs were asked to simulate a bisulfite conversion (changing cytosines to thymines) and to predict the effects on amino acid sequences of non-synonymous polymorphisms (changing nucleotides in different positions of codons). Results from the LLMs were evaluated manually by a human expert.

For the retrieval of orthologs in mouse and the identification of gene ontologies (biological process) and KEGG pathways, randomly selected lists of human genes were used as inputs for the six LLMs. The DAVID [35] and g: Profiler [36] tools were used for the confirmation of orthologs, and the DAVID [35], AmiGO [39], and KEGG databases [37] were employed to confirm the main gene ontologies and KEGG pathways.

A Volcano plot was generated in R Studio (version 2024.12.0) for a synthetic dataset of gene expression (we did not use previously published gene expression figures, as some LLMs might have them in their training). The six LLMs were asked to interpret the Volcano plot, and their results were compared with the analysis by a human expert (the Volcano plot showed 7 upregulated genes and 3 downregulated genes). The six LLMs were asked to write an R script to generate a Volcano plot for a dataset of gene expression as input. RStudio [40] was employed manually to run the R scripts automatically generated by the tested LLMs and to generate the respective visualizations of synthetic gene expression data. All the prompts [41, 42] used in this study are provided in Supplementary File **S1**.

Taking into account the exploratory nature of these analyses [43], we used convenience sample sizes and descriptive statistics and focused on detailed examples of the observed confabulations. Percents, or fractions, of correct answers (accuracy) [44] were identified for each tool, and selected confabulations were extracted. Averages of accuracy were calculated for each LLM and for each task (for those tasks with answers for all 6 LLMs).

## 3 RESULTS

**Fig. (1):**
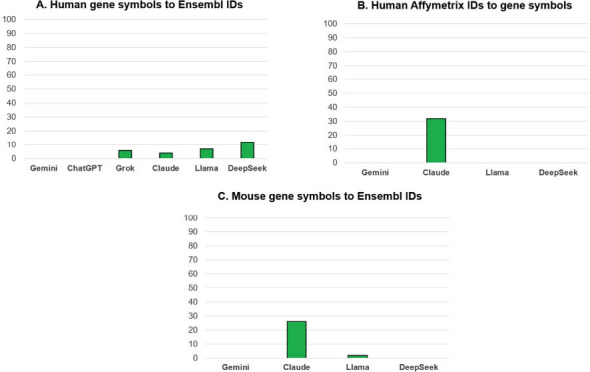
Accuracy of the six free LLMs tested in three types of bioinformatics tasks for conversions of identifiers in genomics. **A**: Conversion of human gene symbols to Ensembl gene IDs. **B**: Conversion of human Affymetrix IDs to human gene symbols. **C**: Conversion of mouse gene symbols to Ensembl gene IDs. All numbers are percent of accuracy. These results show that the accuracy for the three ID conversion tasks, in general, is very low for the tested LLMs.

**Fig. (2):**
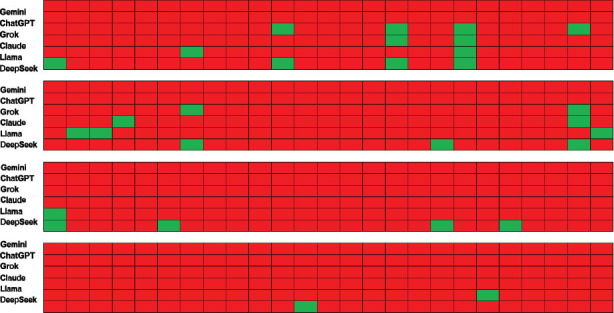
A detailed view of confabulations for six free LLMs, in a simple bioinformatics task (converting human gene symbols to Ensembl gene IDs). Confabulations are shown in red color, and correct results (in comparison to an expert-curated consensus list) are shown in green color. Each one of the 4 blocks shows the results for 25 genes (each column is a gene). These results highlight in detail the high number of mistakes for the conversion of IDs in the outputs of the 6 LLMs tested.

An analysis of the tasks of ID conversions showed a low accuracy, in general, of the tested LLMs, for different types of conversions and in two organisms (Fig. **1**). A detailed visualization of one common type of ID conversion highlighted the large number of incorrect answers for the LLMs (Fig. **2**). Of interest for a better understanding of errors generated by the LLMs, selected examples of confabulations are shown in Table **2**. In the majority of cases, the LLMs provided IDs for other genes and, in some cases, they reported non-existent IDs.

**Fig. (3):**
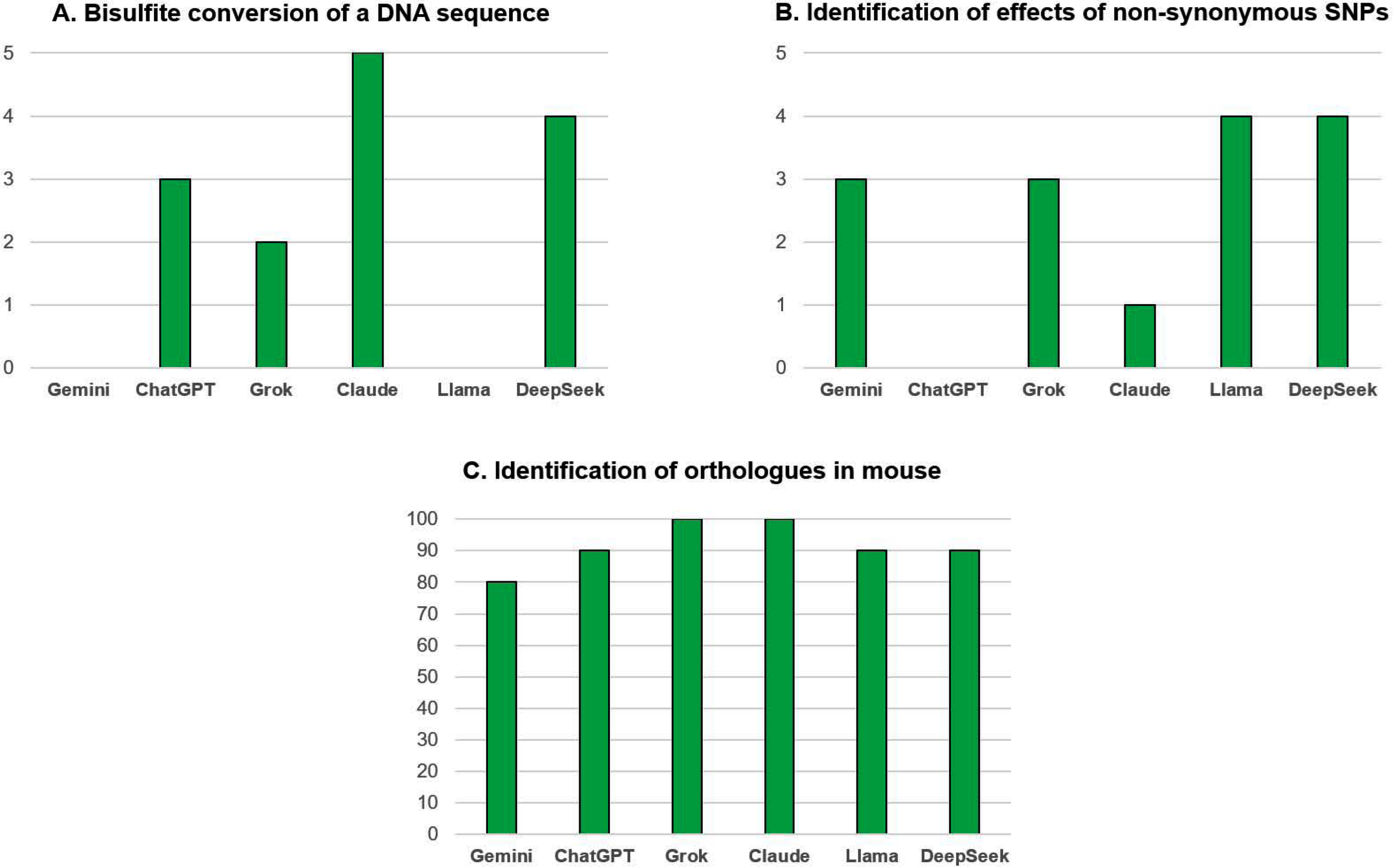
Accuracy of the six free LLMs tested in three types of bioinformatics tasks. **A**: Bisulfite conversion of a DNA sequence. **B**: Identification of the effect of amino acid sequence for DNA polymorphisms. **C**: Identification of orthologues in mouse. Numbers in C represents percents; numbers in A and B are over a total of 5. These results highlight a heterogeneity in the errors produced by the LLMs in the tasks of panels A and B; for the identification of orthologues in mouse the performance of the LLMs was higher.

Regarding DNA sequence manipulation and analysis, some LLMs failed in the simulation of a bisulfite conversion (Fig. **3A**) and in the prediction of changes in amino acids for non-synonymous polymorphisms (Fig. **3B**). Table **3** shows examples of the errors of some of these LLMs, such as the absence of converting some cytosines or providing other types of amino acid changes.

**Fig. (4):**
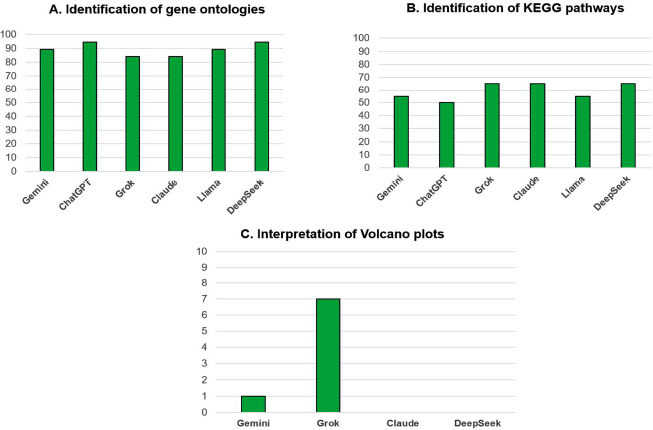
Accuracy of the six free LLMs tested in three types of bioinformatics tasks. **A**: Identification of gene ontology (Biological process) for a list of genes. **B**: Identification of KEGG pathways for a list of genes. **C**: Interpretation of a Volcano plot of gene expression. Numbers in A and B represents percents; numbers in C are over a total of 10 differentially expressed genes. These results highlight a low level of performance for the tested LLMs in the tasks of identification of KEGG pathways and in interpretation of Volcano plots; for the task of the identification of gene ontologies the observed performance was better.

For the identification of orthologs in mouse (Fig. **3C**) and for the retrieval of gene ontologies (Fig. **4A**), the LLMs worked well in general. However, for the retrieval of KEGG pathways, several LLMs showed a lower level of performance (Fig. **4B**). A detailed analysis identified that some LLMs generated answers that are not coherent with defined KEGG pathways (Table **3**).

The test for the interpretation of complex graphs of gene expression found that several LLMs failed to provide a correct interpretation of the Volcano plot **(**Fig. **4C**), and a detailed analysis found that some LLMs reported genes that were not included in the analyzed plot (Table **3**).

**Fig. (5):**
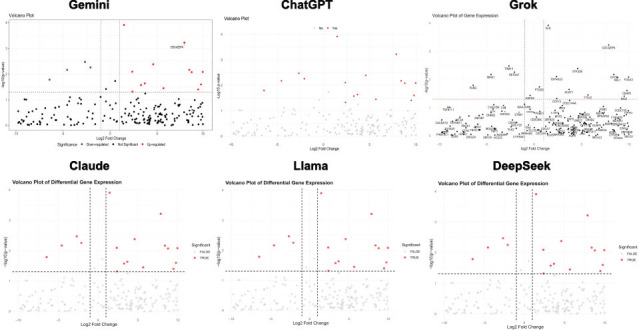
An overview of Volcano plots resulting from the automatically generated code by the six free LLMs. These results highlight that all the six LLMs generated working R code (with a single prompt), with differences in the amount of annotation shown in the Volcano plots.

The analysis of the automatically generated R scripts showed that all of them worked: they ran without problems, producing the expected figures. Fig. **5** shows the diversity of the Volcano plots generated by each LLM, with some of them showing more detailed information.

In terms of averages of accuracy, the LLM with the best performance (for 7 tasks) was DeepSeek (75%). In terms of tasks (for the 6 LLMs), the automatic generation of R code showed the best performance (100%, with a single prompt), and the conversion of human gene symbols to Ensembl gene IDs exhibited the worst performance (5%).

## 4 DISCUSSION

This is one of the first analyses of the performance of several available LLMs for a number of multiple bioinformatics tasks, with varying degrees of complexity. Our analysis identified a multiplicity of confabulations for different types of results generated by the tested LLMs, highlighting the need for the use of LLMs specifically focused on bioinformatics [32-34, 45, 46]. Of particular relevance, code created by LLMs might be useful to lower the entry barrier for the development of advanced computational methods and prototypes [45, 47-49] -a concept recently called vibe coding [50]-, in addition to serving as a teaching tool for wet lab scientists [40, 51-55].

A recent article by Smith *et al.* [56], published in November 2023, proposed that the term confabulations might be better used for referring to the inaccurate outputs of LLMs. The authors mentioned that hallucinations implied the existence of sensory perception and consciousness by the LLMs and that confabulations better reflect the context-dependent generation of inaccurate content by LLMs [56]. A recent Nature paper, published in June 2024, by Farquhar *et al.* [57] also proposed the use of the confabulation term, mainly to differentiate from other types of errors generated by LLMs, such as those arising from training on erroneous data. In a recent preprint, Rawte *et al*. [58] proposed some categories for the severity of LLM hallucinations (mild, moderate, and alarming) and six different types: Numeric nuisance, Acronym ambiguity, Generated golem, Virtual voice, Geographic erratum, and Time wrap, in addition to two primary orientations: Factual mirage and Silver lining.

The potential causes of the confabulations found here might be multiple, such as those related to the quality of the training datasets [59], the autoregressive nature of LLMs [59], and the lack of effective use of existing information [60], among others.

In a 2024 preprint about an exploratory study, Yin *et al*. [24] tested several LLMs (such as GPT-3.5, GPT-4, Llama 2, and Google Bard) for 6 bioinformatics tasks, such as identifying coding regions in a DNA sequence, recognizing potential gene and protein names in sentences, and generating answers to a bioinformatics problem set. They found differences in the performance between the LLMs and observed several mistakes in the answers provided by the platforms. In another recent preprint, Mondillo *et al*. [61] explored the use of ChatGPT4o as a guide for non-specialist researchers for complex bioinformatics processes (particularly on research on allergens). In a preprint, Wang *et al*. [23] employed ChatGPT Plus to generate interpretations of figures from omics research, such as Volcano plots for gene expression, and found that the LLM can generate explanations but that it struggled with quantitative analysis of visual elements.

In a recent article, Tang *et al*. [45] developed BioCoder as a benchmark to evaluate LLMs in creating bioinformatics-specific code. They tested several LLMs, including GPT-3.5-Turbo and GPT-4, and found a better performance for the larger models. In a preprint, Dong *et al*. [33] shared the development of BioMANIA, an open bioinformatics pipeline, natural language-oriented, and AI-driven. The authors highlighted that tool-specific LLMs might have a lower number of confabulations in comparison to general-purpose LLMs [33]. In a recent article from the Ten Simple Rules series, Lubiana *et al*. [51] proposed recommendations for harnessing the power of LLMs in computational biology, which include using them for improving code readability and documentation.

A major issue for the use of LLMs in research tasks is the “Black Box” phenomenon [62], as information about the datasets used for their training is not transparent. It is possible that the paid versions of these LLMs might have a better performance, in comparison to the mini and free versions tested here. In terms of the importance of having high-quality information for scientific activities (which is negatively affected by the presence of incorrect data, such as confabulations), several recent articles have explored and discussed the use of LLMs for several tasks (in areas such as epidemiology, as an example) [5, 63-69], highlighting the need for further improvements for adequate use in research procedures.

Recently, Lobentanzer *et al*. have highlighted that the use of several available LLMs, created by companies, is challenged by the fact that many of them do not provide detailed information on their algorithms and are subjected to major commercial pressures [70]. They also highlighted the importance of objective and neutral comparisons of the performance of available LLMs [70].

Limitations of the current study include the fast changes in the capabilities and outputs of the LLMs [71], which are showing major improvements within weeks, and the exploratory nature of these comparisons, which might be the basis for future analytical and deeper examinations.

Future studies will be needed for a better understanding of the causes of confabulations of LLMs in research-related tasks [71] and for testing LLMs in more complex and challenging bioinformatics tasks [34, 60, 70, 72-74]. In addition, the implementation of research guidelines for bioinformatics tasks, similar to the MI-CLAIM-GEN [75], will be quite useful. Finally, there is a major need for further research on generative AI and health sciences carried out in the Global South [70, 76].

## CONCLUSION

The current study compared the performance of the free versions of six LLMs (Gemini, ChatGPT, Grok, Claude, Llama, and DeepSeek) in a number of tasks commonly used in bioinformatics and computational genomics, with varying levels of difficulty. We found a high number of errors (confabulations) in the output from these LLMs, and we identified a potential use for automatic generation of computer code.

## Figures and Tables

**Table 1:** Overview of the six freely available LLMs tested in this study.

**LLMs**	**Model Used**	**Company**	**Option to Upload Files**	**URL**
Gemini	1.5 Flash	Google	Images	gemini.google.com
ChatGPT	4o Mini	OpenAI	Yes	chatgpt.com/
Grok	Grok 2	xAI	Yes	x.com/i/grok
Claude	Claude 3.5 Sonnet	Anthropic	Yes	claude.ai/new
Llama	Llama 3	Meta	No	meta.ai
DeepSeek	V3	DeepSeek	Yes	chat.deepseek.com

**Table 2:** Selected examples of the distinct types of confabulations generated by six free LLMs in three types of bioinformatics tasks for conversions of identifiers in genomics. Upper part: Conversion of human gene symbols to Ensembl gene IDs. Middle part: Conversion of mouse gene symbols to Ensembl gene IDs. Lower part: Conversion of human Affymetrix IDs to human gene symbols. These selected examples highlight cases in which the LLMs provide IDs that correspond to other genes or that do not exist.

**Human Gene Symbol**	**LLM/Response**	**Verification**
*ACAT1*	Gemini/ENSG00000138668	It is the ID for *HNRNPD*
*ZNF267*	Grok/ENSG00000169194	It is the ID for *IL13*
*TSPAN16*	Claude/ENSG00000177697	It is the ID for *CD151*
*ANKS6*	ChatGPT/ENSG00000108779	It does not exist
*MAP1LC3C*	DeepSeek/ENSG00000162339	It does not exist
*SPNS3*	Llama/Not found	ENSG00000182557 is the ID
**Mouse Gene Symbol**	**LLM/Response**	**Verification**
Adamts17	DeepSeek/ENSMUSG00000031977	Retired from Ensembl
Cars2	Claude/ENSMUSG00000025958	It is the ID for Creb1
Nadsyn1	Llama/ENSMUSG00000068744	It is the ID for Psrc1
**Human Affymetrix ID**	**LLM/Response**	**Verification**
207123_s_at	Gemini/RPL7	*MATN4* is the correct gene
214955_at	Llama/ZNF768	*TMPRSS6* is the correct gene
206943_at	DeepSeek/CXCR4	*TGFBR1* is the correct gene

**Table 3:** Selected examples of the distinct types of confabulations generated by six free LLMs in five bioinformatics tasks (from top to bottom): Bisulfite conversion of a DNA sequence; Identification of the effect of amino acid sequence for a DNA polymorphism; Identification of gene ontology (Biological process) for a list of genes; Identification of KEGG pathways for a list of genes; Interpretation of a Volcano plot of gene expression. These selected examples highlight cases in which the LLMs give incorrect answers, such as providing other DNA sequences or types of amino acid polymorphisms, in addition to assigning incorrect GOs or KEGG pathways. For the analysis of Volcano plots, the selected examples show that some LLMs mention genes that are not present in the figures.

**DNA Sequence**	**LLM/Response**	**Verification**
TAC GCG ATC GTG GCA ATT	Gemini/TAT GCG ATC GTG GGA ATT	TAT GTG ATT GTG GTA ATT is correct
ACG CGG GCA CGG CTG TAA	Llama/ACG CGG GTA CGG CTG TAA	ATG TGG GTA TGG TTG TAA is correct
**DNA polymorphism**	**LLM/Response**	**Verification**
GTG to ATG	ChatGPT:Glutamic acid/Lysine	Valine/Methionine is correct
TCC to TAC	Claude:Proline/Glutamine	Serine/Tyrosine is correct
**Gene to Gene Ontology**	**LLM/Response**	**Verification**
*ZNF804B*	Llama/Transcription regulation	*ZNF804B* does not have a GO (BP)
*INPP4B*	Gemini/ Cellular lipid metabolic process	It is not a GO (BP) for *INPP4B*
**Gene to KEGG Pathway**	**LLM/Response**	**Verification**
*TUBA1A*	ChatGPT/Microtubule dynamics	It is not a KEGG pathway for TUBA1A
*MMP12*	Grok/ECM-receptor interaction	*MMP12* does not have a KEGG pathway
**Volcano Plot**	**LLM/Response**	**Verification**
-	Gemini/TRIM15 and RETSAT as upregulated	TRIM15 and RETSAT were downregulated
-	Claude/STX2B and TRIM37 as differentially expressed	Those genes do not appear on the Volcano plot
-	DeepSeek/TBM18 and STIC10 as differentially expressed	Those genes do not appear on the Volcano plot

## Data Availability

The authors confirm that the data supporting the findings of this research are available within the article.

## LIST OF ABBREVIATION

LLMs: Large Language Models
