# Supplementary material for: Multiple Confabulations Found in Bioinformatics Tasks Carried Out by Several Free Large Language Models
Source: Curr Genomics. 2026 Mar 19;26(6):703–12. doi: 10.2174/0113892029403403260112075225 (PMC13154259; doi:10.2174/0113892029403403260112075225)
Supplement: Supplementary file 1 [file CG-26-6-703_SD1.pdf]

Supplementary Material

Multiple Confabulations Found in Bioinformatics Tasks Carried Out by Several Free Large Language Models

Diego A. Forero[1,*]

[1]*School of Health and Sport Sciences, Fundación Universitaria del Área Andina, Bogotá, Colombia*

Forero DA. Multiple Confabulations Found in Bioinformatics Tasks Carried Out by Several Free Large Language Models.

Supplementary File S1

Prompts used, with six LLMs, in this study

Conversion of identifiers in genomics
Please convert the following human gene symbols to Ensembl gene IDs
Please convert the following Affymetrix IDs to human gene symbols
Please convert the following mouse gene symbols to Ensembl IDs

Bisulfite conversion of DNA sequences
Please do a bisulfite-conversion for the following DNA sequences:

Prediction of changes in amino acids for non-synonymous polymorphisms
What are the functional annotations for the single nucleotide polymorphisms in the following human coding DNA sequences?

Identification of orthologues in mouse
Please identify the orthologues in mouse for the following list of human genes:

Identification of gene ontology (Biological process) for a list of genes
Please identify the respective main Gene Ontology (Biological Process) for the following genes:

Identification of KEGG pathways for a list of genes
Please identify the enriched main KEGG Pathway for the following genes:

Interpretation of a Volcano plot of gene expression
Please interpret the following figure of gene expression data:

Automatic writing of R code to generate Volcano plots of gene expression data
Please write a simple R script to generate a volcano plot for a dataset (with a csv. file as input) of genome-wide expression for 1000 genes. The dataset has three columns (gene_symbol, log2fc and pvalue).
